# Robust Korean License Plate Recognition Based on Deep Neural Networks

**DOI:** 10.3390/s21124140

**Published:** 2021-06-16

**Authors:** Hanxiang Wang, Yanfen Li, L.-Minh Dang, Hyeonjoon Moon

**Affiliations:** Department of Computer Science and Engineering, Sejong University, Seoul 143-747(05006), Korea; hanxiang@sju.ac.kr (H.W.); 1826535091@sju.ac.kr (Y.L.); minhdl@sju.ac.kr (L.-M.D.)

**Keywords:** Korean license plate recognition, image preprocessing, deep learning

## Abstract

With the rapid rise of private vehicles around the world, License Plate Recognition (LPR) plays a vital role in supporting the government to manage vehicles effectively. However, an introduction of new types of license plate (LP) or slight changes in the LP format can break previous LPR systems, as they fail to recognize the LP. Moreover, the LPR system is extremely sensitive to the conditions of the surrounding environment. Thus, this paper introduces a novel deep learning-based Korean LPR system that can effectively deal with existing challenges. The main contributions of this study include (1) a robust LPR system with the integration of three pre-processing techniques (defogging, low-light enhancement, and super-resolution) that can effectively recognize the LP under various conditions, (2) the establishment of two original Korean LPR approaches for different scenarios, including whole license plate recognition (W-LPR) and single-character license plate recognition (SC-LPR), and (3) the introduction of two Korean LPR datasets (synthetic data and real data) involving a new type of LP introduced by the Korean government. Through several experiments, the proposed LPR framework achieved the highest recognition accuracy of 98.94%.

## 1. Introduction

As one of the convenient and comfortable means of transportation, the number of motor vehicles has rocketed in recent years. Although the benefits of using private cars are undeniable, it causes a huge burden on traffic control and vehicle management. As the license plate (LP) is the only way to identify a vehicle, automatic license plate recognition (LPR) systems have brought significant changes to traditional vehicle management. These systems can detect and recognize LP automatically with high accuracy, so they help to reduce the involvement of humans and further facilitate automatic parking or traffic management.

Several years ago, a new type of LP was introduced by the Korean government that facilitates more effective management of different types of vehicles [1]. A detailed description of the new Korean license plate system is shown in Table 1. There are two main categories of vehicles in South Korea, including private vehicles and commercial vehicles. The common LP format of private vehicles is made up of two initial digits, one Korean character, followed by four digits. On the other hand, the LP format consists of two lines, province or city name plus two digits on the first line, one Korean character, and four digits on the other line for commercial vehicles. However, the taxi LP is different in format, which incorporates all information into one line.

Recently, many researchers have applied deep learning methods to LPR [2,3,4]. For existing systems, some standard pre-processing methods based on OpenCV were used to deal with the low-quality images. After that, there are three common processes for the LPR, including license plate detection (LPD), character segmentation, and recognition. However, the environment has negative effects on the result of the LPR system. For example, during foggy weather, the scattering effect of atmospheric particles leads to severe degradation of the image quality. The brightness and contrast of images captured at night are low. Besides, the collected data is blurred due to the bad acquisition system. Due to the influence of many external conditions, LPR technology has not been mature and stable [2,3]. Some specific processing technologies, such as defogging, low-light enhancement, and super-resolution, are used to deal with the images under complicated backgrounds [5,6]. A novel LPR system with a robust performance was proposed in this study, which integrates computer vision (CV), image processing, and deep neural network technology.

Currently, there are some existing limitations for LPR systems. Firstly, various external conditions often affect the results of LPR in real life. Moreover, the LP of every country has its own characteristics, such as colors, format, size, and letters. Besides, a large dataset is required to achieve good recognition accuracy by using deep learning methods, but the data of various Korean characters are not easy to be obtained. By analyzing the limitations of the current LPR system, the main contributions of this research are listed as below:

1. The LPR system deals well with images under various conditions, such as low light, foggy, and low-resolution images.
2. Two new Korean LPR models are proposed to perform the recognition of both numbers and Korean characters.
3. The processing speed of the LPR system (including vehicles detection stage, LPD stage, and LPR stage) can meet the needs of real-time computation, which can be integrated into live applications.
4. The collection of a large Korean license plate dataset that contains both real LP images and synthetic LP images.

This paper is arranged as follows. Section 2 illustrates some recent work that is related to the topic of LPR. The collected dataset is mentioned in Section 3. Section 4 describes the proposed LPR system. Some experimental results are shared and discussed in Section 5. In Section 6, the final conclusions for this study are drawn. 

## 2. Related Work

Currently, the major means of transportation are vehicles, including cars, trucks, and buses, which are an indispensable part of daily life. To facilitate the management of vehicles, some related smart technologies have been developed, such as vehicle detection and tracking. In particular, LPR has become a high-profile research topic among many researchers in the computer vision field. For example, Selmi et al. proposed a deep learning-based LPR system [3]. Although the system performed well in both ideal and severe conditions, it only supported the recognition of numbers and English letters and did not involve the recognition of characters. Similarly, a robust system was created to recognize the LP with numbers and letters by using CNN, which was capable of working with various LP templates under different environmental variations [2]. In other work, an LPR system, including the recognition of numbers and Korean characters, was developed to recognize the images obtained from the vehicle black box. Experiments showed the overall accuracy is 89.79% by using a classifier called the k-nearest neighbor. The probable cause was that the data was so small that the classifier cannot acquire enough features [7]. As for Chinese LP character recognition, a kind of feature extraction method called coarse-to-fine was applied to design the CNN architecture, and the experimental result proved the accuracy of the proposed method is 98.7% that was much better than the Back Propagation (BP) model and SVM classifier. Although Chinese characters are recognized in the experiment, Chinese characters only account for 13% of the training data that causes imbalanced data problems [4]. Besides, a framework was designed to detect the LPs from multiple directions, which can improve the performance of LPD when the obtained LP images are rotated [8]. Nevertheless, the developed method was not an end-to-end model to simultaneously handle detection and recognition tasks. In another work, the problem of LPD and LPR was tackled well by using a unified model, while the processing speed needs to be further improved [9].

Moreover, LPR has been applied to many fields, such as urban surveillance, parking management, and intelligent transportation. A vehicle attendance monitoring system with a high practical value was developed by correctly identifying the LP [10]. A smart parking management system was implemented to improve the inefficient management problems left by the traditional parking management, in which LPR plays a great role [11]. A smart camera for transportation security and law enforcement applications was proposed to identify the parameters of cars, such as make, model, color, and LP [12]. Based on LPR, there is a commercial software: OpenALPR [13]. The software can perform well, but the exact detail of the model is not available.

Considering LPR is still a tricky problem because of some restrictions, such as severe foggy weather, weak light environment, and low-quality images, some researchers provided various solutions to deal with the low-quality images. An LPR system applied filters and morphological processing for segmentation, and a BP network for recognition, which can not only tolerate the noise of 20% but also achieve an 85% recognition rate [14]. In other work, an algorithm called dark channel prior [15] was applied to deal with the images under foggy weather in the LP detection process, and experiments showed that the information of the LP can be restored well [6]. Besides, a super-resolution algorithm was used for the LPR system, which can effectively enhance the identification accuracy and robustness under complicated backgrounds [5].

In this study, a system integrated with image preprocessing technologies is proposed to recognize Korean LPs, which can be applied for smart parking or other fields. Besides, two different methods are proposed in the LPR process. In terms of recognition accuracy, the proposed methods outperform the state-of-the-art LPR system by using the same dataset.

## 3. Dataset Acquisition

### 3.1. The Proposed Dataset

In this study, two different frameworks based on deep learning are proposed to recognize the Korean LP characters. The training of the deep learning model needs the support of huge data to get better recognition results. However, it takes lots of effort to collect and validate real Korean LP images manually. Thus, in this study, large synthetic LP images were generated by a python script, along with real LP images were shot outdoor by a mobile phone. Different from the previous related research data, the proposed dataset not only has a large amount but also involves a new type of LP introduced by the Korean government.

**Figure 1:**
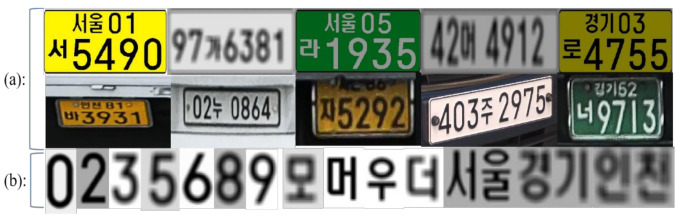
Sample images from two sets of data, which include (**a**) whole LP images and (**b**) single-character images.

There are two sets of data used for different LPR methods. The recognition is based on the whole LP for the whole license plate recognition (W-LPR) approach, and every image of the dataset is the whole LP. 500,000 synthetic LP images and 10,500 real LP images were added to the training process. Both synthetic LP images and real LP images size are 128 × 64. To improve the diversity of the data samples, the brightness, contrast, noise, and other variables in the image are changed to make the synthetic data more diverse and realistic. The recognition is based on the character for the SC-LPR approach, and there are 67 types of characters separated from LPs in the dataset, including 10 kinds of numbers, 40 kinds of Hangul, and 17 kinds of region names, as shown in Table 1. The amount of the dataset is 216,500, and all images were saved as 32 × 32. In Figure 1, the synthetic Korean LP images (first line) and the real Korean LP images (second line) in the first set of data are shown in (a). The number, Hangul, and region name (third line) in the second set of data are shown in (b).

### 3.2. Other Datasets

In this study, the proposed system is compared with the state-of-the-art by using the same dataset. The dataset named Korean car plate (KarPlate) dataset is available to download. The KarPlate dataset includes LPD, LPR, and EER subsets, which are used for license plate detection, license plate recognition, and end-to-end recognition (EER), respectively. For the LPD and LPR subsets, there are 3417 images in the training set and 850 images in the testing set. For the EER subset, there are 929 images in total.

## 4. Methodology

**Figure 2:**
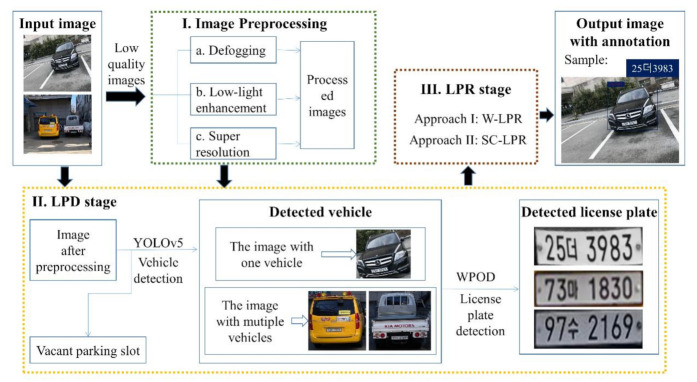
The flowchart of the LPR system from the input image to the labeled output image is divided into three parts, which include image preprocessing, LPD, and LPR.

As shown in Figure 2, the flowchart of the LPR system from the input image to the labeled output image is divided into three parts, including image preprocessing, LPD, and LPR. Firstly, the input image with the vehicle is obtained from CCTV. Three image processing technologies, defogging, low-light enhancement, and super-resolution are used to avoid the influence of bad environments on the recognition results (see Section 4.1 for details). In the LPD stage, YOLOv5 [16] is used to detect vehicles in the early stage of license plate detection. At the same time, the vacant parking slot is detected in this stage. Considering that the LPs in most vehicle images are slanted, the Warped Planar Object Detection Network (WPOD-NET) [17] can correct and detect the slanted LPs in the images, so it is used to detect the LP area. The specific steps for LP detection are introduced in Section 4.2. After that, two different methods are applied to the last stage of the LPR system, which is introduced in Section 4.3. 

### 4.1. Image Preprocessing

#### 4.1.1. Defogging and Dehazing

During atmospheric phenomena, such as fog and haze, the scattering effect of atmospheric particles leads to severe degradation of the image quality collected by the image sensor [18]. To reduce the influence of these outdoor atmospheric phenomena, a defogging method called the dark channel prior algorithm [15] is applied to process the image before the LPR phase.

Through the analysis of many haze-free images, it is discovered that among the three channels (RGB) of each image, the intensity of some pixels is very low, almost tends to 0 [15]. In the process of defogging, the dark channel image is obtained by filtering the darkest value of the local area. Formula (1) illustrates the calculation method of the dark channel image.

(1) $$D\left(x\right)=\underset{y=\Omega \left(x\right)}{\min}\left(\underset{c\in \left\{r,g,\right.\left.b\right\}}{\min}\left(I^{c}\left(y\right)\right)\right),$$

In Formula (1), D  is the dark channel image, and x is the pixel point in the image. c represents one of the three RGB channels, and $\Omega \left(x\right)$ represents the minimum filtering area centered on the pixel point x. $O\left(x\right)$ is the output image after defogging, and $I\left(x\right)$ represents an input image. If the transmission $t\left(x\right)$ is too small, it will lead to uneven distribution of light and dark areas in the output image. Therefore, $t_{min}$ is used to limit the minimum value of $t\left(x\right)$. 

Formula (2) is the final expression of the defogging algorithm, in which the atmospheric light L and transmission $t\left(x\right)$ are the necessary conditions for calculation. The value of atmospheric light can be calculated through the dark channel prior, and then the transmittance can be further calculated.

(2) $$O\left(x\right)=\frac{I\left(x\right)-L}{\underset{}{max}\left(t\left(x\right),t_{min}\right)}+L,$$

#### 4.1.2. Low-Light Enhancement

To solve the problems of low brightness, low contrast, and blurry images captured at night, a method called contrast limited adaptive histogram equalization (CLAHE) is used to improve image brightness, enhance contrast, restore more details of the image, and achieve a better visual effect [19]. Formula (3) is the expression of the CLAHE algorithm. During the implementation of the algorithm, the value of Cliplimit as a threshold value needs to be set in advance. $P_{Ori}$ and $P_{CLAHE}$ represent the original pixel distribution and the pixel distribution using the algorithm, respectively. Sum refers to the sum of all pixel values of the histogram that exceed Cliplimit. N is used to represent the number of pixels in the image.

(3) $$P_{CLAHE}=\left\{\begin{array}{c} P_{Ori}+\left(Cliplimit-\frac{Sum}{N}\right),\;\;\;\;if\;P_{Ori}>\;\left(Cliplimit-\frac{Sum}{N}\right)\;\;\\ Cliplimit,\;\;\;\;Others\;\;\;\;\;\;\;\;\;\;\;\;\;\;\;\;\;\;\;\;\;\;\;\;\;\;\;\;\;\;\;\;\;\;\;\;\;\;\;\;\;\;\;\;\;\;\;\;\;\;\;\;\;\;\;\;\;\;\;\;\;\;\;\;\;\;\;\;\;\;\;\;\; \end{array}\right.,$$

Each pixel on an image corresponds to a gray level, and the function of the histogram is to count the number of pixels for each gray level. In the darker image, the components of a histogram are concentrated on the side of a low gray level. Histogram equalization can make the image contain all gray levels as much as possible and make it distribute evenly. The main difference between CLAHE and common Adaptive Histogram Equalization (AHE) is its contrast limiting. In the CLAHE, the contrast limiting must be used for each small area. The CLAHE algorithm is mainly used to overcome the problem of the over-amplification noise of the AHE.

#### 4.1.3. Super-Resolution

To improve the resolution of the images captured far from the camera, or by a low-resolution sensor, the most direct way is to improve the optical hardware of the acquisition system, but this approach is limited by the high manufacturing cost. Therefore, from the view of the algorithm, deep learning-based image super-resolution reconstruction is a reasonable approach to deal with low-quality images. Currently, the most important application is to magnify the area of interest (ROI) in surveillance and reconstruct it. For example, presently, CCTV is widely used in various public places. In monitoring, it is necessary to magnify the target of the scene, such as a car license plate. Super-resolution can eliminate noise and blurring caused by optical elements, thus providing more useful information from the data obtained from CCTV. 

Recently, the research of super-resolution has made some progress due to the development of CNN [20]. In particular, Enhanced Super-Resolution Generative Adversarial Networks (ESRGAN) showed great visual effects [21]. The ESRGAN model was mainly modified from two parts of the SRGAN model [22], including the exclusion of Batch Normalization (BN) layers and the replacement of the original basic block. The ESRGAN model includes a generator network and discriminator network. The design of the generator network refers to the residual structure of ResNet, which makes the network deeper and stabilizes the learning process. The generator network consists of 5 convolution layers (3 × 3) with the LeakyReLU activation function and 23 residual modules. On the other hand, the discriminator uses the VGG model that contains 10 convolution blocks. Each convolution block contains a 3 × 3 convolution layer, a BN layer, and a LeakyReLU activation function. It was verified that the ESRGAN model enhanced visual effects and reduced computational complexity significantly because it reconstructed the images with more detailed features and sharper edges. Therefore, the ESRGAN was employed as an image preprocessing to improve the overall perceptual quality before implementing the LPR in this research. This model raised the computational time of the whole system due to its complexity [21], which will be explained in experiments Section 5.1.

### 4.2. License Plate Detection

Inspired by the previous research [16], the YOLOv5 model combined with the PyTorch framework is used to detect vehicles, and then the WPOD-NET model [17] is used to obtain areas of LPs in this study. YOLOv5 is characterized by fast detection speed and high recognition accuracy. After the vehicle is detected quickly, the image is input into the WPOD-NET model to detect the LP area. WPOD-NET model can learn the distortion degree of the LP from various vehicle images to generate the coefficient of regression radiation transformation and then generate the rectangular LP under the front view. The reason why vehicle detection is applied before vehicle license plate detection is that there are multiple LPs in some images, and the WPOD-NET model can only process a single spatial transformation in the input image.

YOLOv5 is the latest improved version of YOLO models, and its main improvement is reflected as follows. Firstly, in the data preparation stage, YOLOv5 uses some data augmentation techniques to improve the generalization ability of the model, and the adaptive method is added to update the optimal anchor box values of the dataset. Secondly, the network structure combined Darknet with Cross Stage Partial Network (CSPNet) [23] in both Backbone and Neck parts. The main purpose of CSPNet is to enable the network architecture to obtain more abundant gradient fusion information and reduce the amount of computation. Moreover, YOLOv5 uses multiple feature maps with different scales. The size of the candidate box for each feature map is different, which improves the model’s ability to recognize small objects [16,24]. The reason why YOLOv5 is selected for vehicle recognition is that it has a fast recognition speed and adaptability to the objects with multi-scales.

The WPOD model contains 21 convolutional layers with a size of 3 × 3 and 18 ReLU activation functions. Four maximum pooling layers with a size of 2 × 2 are used to remove redundant features and reduce the number of parameters. The model uses a residual network structure to increase the depth of the network. A detection block with two convolutional layers is used in the last part of the model to calculate the affine coefficients used to correct the detection area. The WPOD model greatly improves the recognition accuracy of this system.

### 4.3. License Plate Recognition

After the LPD step, two different approaches were proposed to recognize the Korean LP. The first approach (W-LPR) refers to the usage of a deep learning model (CNN + RNN) to train the whole LP images directly. On the other hand, the second LPR method named SC-LPR is based on character recognition, and it contains two main steps. First of all, each character was segmented from the LP, and then the extracted character images (number, Hangul, and region name) were fed into a specific CNN model to perform recognition.

#### 4.3.1. Whole License Plate Recognition (W-LPR)

**Figure 3:**
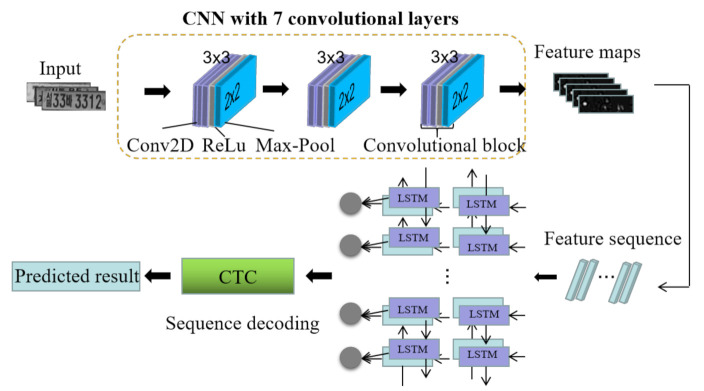
The architecture of the deep learning model for W-LPR. Firstly, a deep CNN is used to extract features and transform them into sequence representation. Secondly, LSTM is trained to obtain context information. Finally, CTC is applied as the last layer of the RNN model to give the final recognition result.

In the W-LPR method, a network Conv7-RNN combining CNN and RNN is proposed to recognize the whole LP without character segmentation and recognition. As shown in Figure 3, the whole deep network model is designed as two parts. In the first part, a custom CNN structure is proposed to extract image features and transform the deep features into sequence representation. In the second part, the special RNN model long short-term memory (LSTM) [25,26] is trained to obtain the context information in the sequence and encode the sequence information. After that, Connectionist Temporal Classification (CTC) [9] was applied as the last layer of the RNN model gives the final recognition result after analyzing the coding result of RNN.

There are two sections in the architecture of the proposed Conv7-RNN model, including CNN and RNN. Inspired by the structure of VGGNet, the CNN section was designed in this study, which consists of 7 convolutional layers of 3 × 3 size and 3 max-pooling layers of 2 × 2 size in three convolutional blocks. There are two convolutional layers and a max-pooling layer in the first and second convolutional blocks, respectively. The third convolutional block has three convolutional layers and one max pooling layer. The number of filters for each convolutional layer is 64, 128, 256, 256, 512, 512, and 512, respectively. The structure of LSTM was applied in the RNN section, which consists of two bidirectional-LSTM layers with 256 hidden units and one CTC output layer. As a special RNN, LSTM can deal with the gradient vanishing problem through a more complex internal structure (memory cell, input gate, output gate, and forget gate) [27]. The bidirectional RNN can train the forward time series and the reverse time series respectively so that the RNN can learn the context information from the series better [28].

#### 4.3.2. Single Character Recognition (SC-LPR)

**Figure 4:**
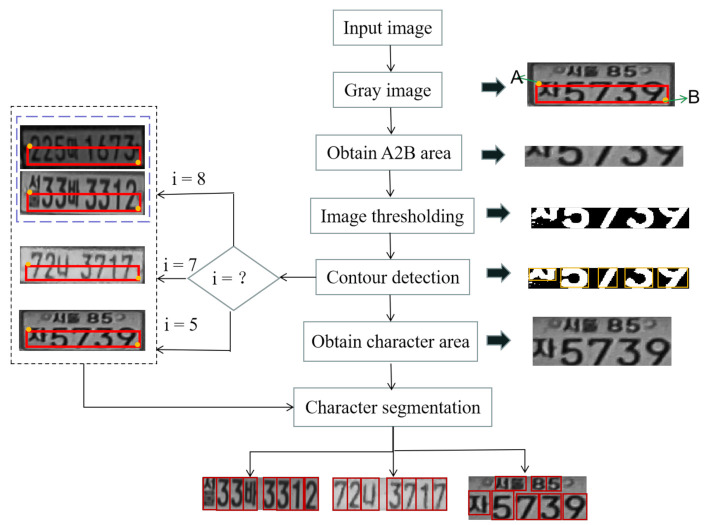
The whole process of character segmentation. ‘i’ means the number of contours. A rectangular area (A2B), which contains a top-left point of A and a bottom-right point B, is obtained to distinguish different types of LPs by measurement.

The character segmentation process of the SC-LPR approach is shown in Figure 4. Firstly, the size of the input image is set to 240 × 80. Since the recognition accuracy of a character depends on the segmentation of the characters on the LP, the noise and redundant blank areas on the LP image should be removed to obtain a better segmentation effect [29]. Before that, the width and height of character areas of different types of LPs need to be calculated. By observing and measuring various LPs, different LP types can be distinguished according to the number of characters in a rectangular area (A2B) with a top-left point of A (15, 48) and a bottom-right point of B (75, 225). We get A2B from the original image, and transform it into a binary image, and use an adaptive threshold algorithm to remove some noise caused by illumination and lighting conditions. After that, the contour detection algorithm is used to detect the contour of all characters on the image, and then reorder all contours from left to right. The first character and the last character can be used to determine the abscissa of the left and right boundaries for the entire character region. However, sometimes the left edge of the first character and the right edge of the last character cannot be directly used as the left and right boundaries of the whole character region. For example, when 7 or 1 is the beginning and end character, it needs to be recalculated. The formula for calculating the abscissa of the left and right boundaries of the character area is as follows:

(4) L,R=I x1+w12−c+15, x2+w22+c+15.

In Formula (4), L and R represent the abscissa of the left and right boundary of the character region, respectively. I refers to the input image, and c is used to represent half the width of a complete character. $x_{1}$ and $w_{1}$ are the abscissa of the left edge for the first character and the width of the character, respectively. $x_{2}$ and $w_{2}$ are the abscissa of the right edge for the last character and the width of the character, respectively. The constant refers to the 15 pixels on the left that are removed when obtaining A2B. After calculating the abscissa of the left and right boundaries, the character region can be obtained. Finally, a character can be segmented according to the proportion of characters on different LPs.

In the process of character recognition, a customized lightweight deep learning framework is proposed. Table 2 shows the basic structure of the model that consists of three convolutional blocks and two fully connected layers. Each convolution block contains a convolutional layer, an activation layer (ReLU), and a max-pooling layer. The kernel size in each convolutional layer is set to 3 × 3, and the step size is 1. The input image of the model is an RGB image with a size of 32 × 32. The softmax function is used to calculate the probability for 67 types of characters.

### 4.4. License Plate Recognition System

**Figure 5:**
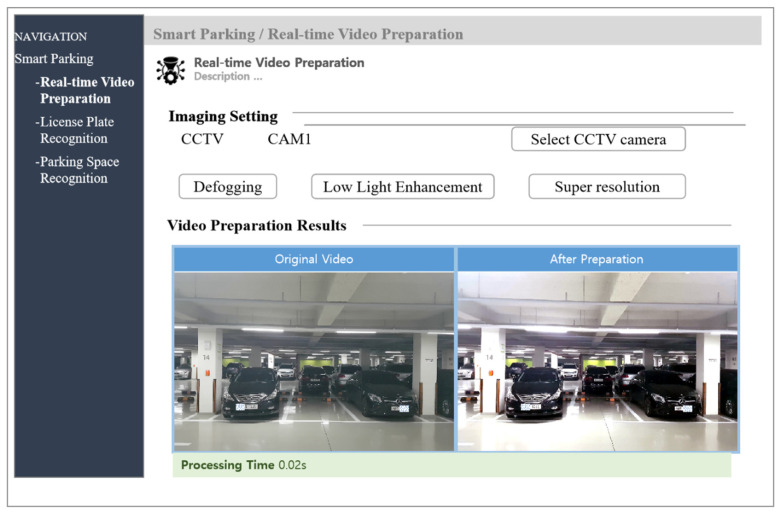
The graphical user interface of the proposed system.

The proposed LPR system is web-based, and the graphical user interface is shown in the Figure 5. There are three interfaces, including image preprocessing, parking space detection, and LPR interfaces. The input data is selected from different CCTV cameras. In the image preprocessing interface, three technologies are used to improve the image quality. The LPs are recognized, and then the available slot is detected in the corresponding interface.

## 5. Experimental Results

In this study, the experiments have been performed on Keras version 2.2.4 using python 3.5. The implementation environment is on a Linux machine pre-installed with Ubuntu 16.04. It has four Titan X 12GB GPUs, an Intel^®^ Core i7-5930K processor, and 64 GB of DDR4 RAM.

### 5.1. Image Preprocessing

**Figure 6:**
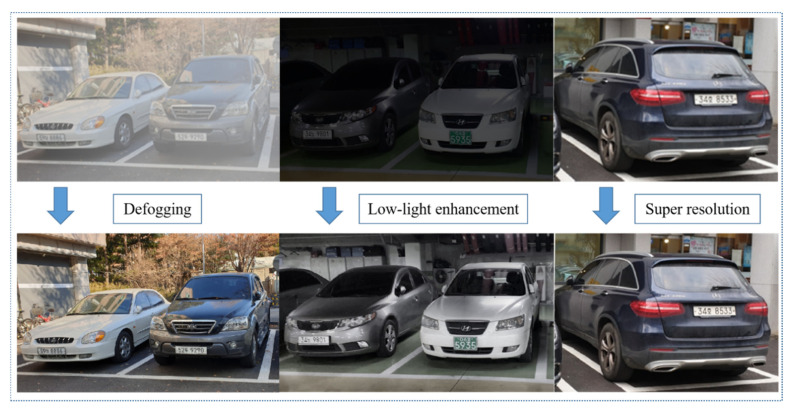
The visual effect of three image preprocessing technologies, which include defogging, low-light enhancement, and super-resolution.

To improve the final recognition accuracy, three image preprocessing technologies were applied at the beginning. This section not only shows the visual effect and the speed of these image processing methods but also demonstrates the influence of image preprocessing on performance. As shown in Figure 6, three image preprocessing technologies were used to improve the quality of the input images. 

Before image preprocessing, the size of all images is 240 × 80. As shown in Table 3, the dark channel prior algorithm used for the defogging process can effectively remove the influence of dust and fog on the image. The low-light enhancement technology, which uses the CLAHE algorithm, can effectively enhance the contrast of the image and increase the image visibility. Besides, after super-resolution, the reconstructed image provides more information and achieves a good visual effect. The experiments show the processing time of fog removal and weak light enhancement is very short for a single image, but the processing speed of super-resolution is relatively slow due to the complicated network architecture. As for the recognition results, it shows that there are many cases that the character was mistakenly identified as other characters before image processing. To distinguish the incorrect results from the correct results, the wrong character is marked in red. As the character segmentation is lacking in the case of blurry images, the recognition results for the whole license plate are better than the recognition results based on separated characters. Nevertheless, whether it is based on the whole license plate recognition or character recognition, most of the characters can be recognized correctly after image processing.

### 5.2. Whole License Plate Recognition (W-LPR)

As mentioned before, the recognition of the LP based on the W-LPR method does not rely on character recognition. Firstly, the DL model requires the whole image to be the input, and the deep features are extracted by CNN, then the local features are gathered by RNN for the sequence recognition. To obtain a model that better fits the dataset, the structure of VGGNet [30,31], was referred to design the proposed CNN section, and then the training performances of different networks with different depths were analyzed based on the synthetic and real datasets. The experimental networks consist of CNN and RNN, where the structure of the RNN section is the same. To verify the effectiveness and significance of convolutional layers in feature extraction, all networks were set with the same hyper-parameters, and the numbers of convolution layers are set as 7, 10, 13, and 16, respectively. In the training process, the initial learning rate is 0.02 and then was gradually optimized according to the change of training gradient. The early stopping approach is used to efficiently monitor the model’s training progress. When the training loss remains stable for a consecutive number of epochs (5), the training process is terminated, and the best training weight is saved. The networks were trained with an optimizer called ADADELTA, and the CTC loss function. During the training process, 500,000 synthetic images were fed into the models to make them achieve a better convergence effect, and then the models were fine-tuned with the real LP images.

**Figure 7:**
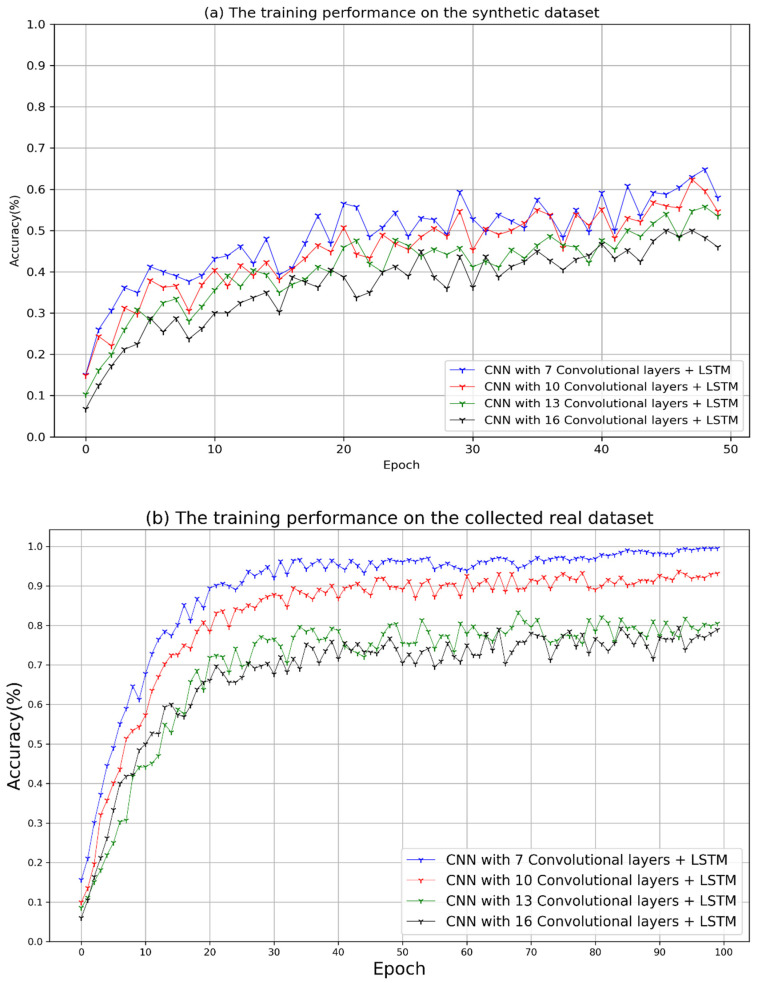
The performance of the whole LP recognition, which includes (**a**) the performance on the synthetic data and (**b**) the performance on the collected data.

As shown in Figure 7a, it shows the recognition accuracy for each network is slowly increasing when training the synthetic dataset, and there is a downward trend at the end of the training. It can be observed that the performance of a network with 7 convolutional layers (Conv7-RNN) is similar to a network consisting of 10 layers, which is a little better than the other networks. To avoid the over-fitting problem, the synthetic data was trained on 50 epochs to obtain the initial model, and then the real data was trained on 100 epochs to fine-tune the parameters of the initial model. Figure 7b indicates the performances of the fine-tuned models on the real dataset. The Conv7-RNN model achieved the highest accuracy of 99.5% at the 72nd epoch. According to the statistics for training time, the whole training process of Conv7-RNN took about 8.3 h, which costs the shortest time among the compared networks. Since the complexity of the network depends on the training time, the network with 16 convolutional layers took the longest training time, which is about 16.5 h. Experiments show the computational complexity of the network increases, and the accuracy of recognition decreases by adding more convolution layers to the network. This is because the network does not have enough neurons to analyze the features extracted from many convolution layers so that the network has a bad generalization effect.

### 5.3. Single Character Recognition (SC-LPR)

In this section, the result of character segmentation is evaluated, and then the performance of character recognition is shown from the view of accuracy and time. Since the effect of character segmentation directly determines the result of character recognition, character segmentation is a vital step in the whole process. 100 real LP images were selected randomly from the collected data to test the effect of character segmentation. Here, the accuracy of character segmentation is defined as below:

(5) $${\mathrm{Accuracy}}_{\mathrm{segmentation}}=\frac{\mathrm{Number}\;\mathrm{of}\;\mathrm{correctly}\;\mathrm{segmented}\;\mathrm{characters}}{\mathrm{The}\;\mathrm{number}\;\mathrm{of}\;\mathrm{all}\;\mathrm{characters}}\;\times \;100\%\;.$$

**Figure 8:**
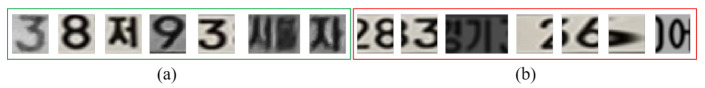
The results of character segmentation, including some successful cases (**a**) and some failure cases (**b**).

A total of 750 characters are segmented from 100 different LP images, of which 745 characters are well segmented. According to the accuracy formula, the segmentation accuracy can achieve 99.3%. The segmentation result for each character is validated manually, as shown in Figure 8, it succeeded in segmenting most of the characters, but there are also some failure cases where the character is segmented incorrectly.

**Figure 9:**
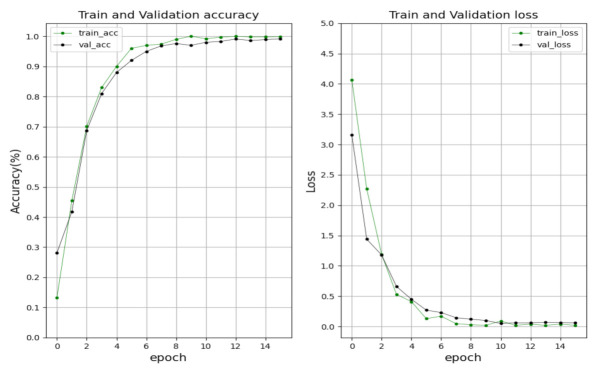
The training and validation performance (accuracy and loss) of the character recognition module.

As for character recognition, 67 kinds of characters were added to train the model, including numbers, Hangul, and region name. In the training process, the basic learning rate and momentum are set as 0.001, 0.9, respectively. The stochastic gradient descent (SGD) method was used to optimize the model. The batch size is 128, and the total epoch was set as 15 times. The performance of training and validation are shown in Figure 9. The training and validation accuracy keep rising, and at the 12th epoch, the accuracy is up to the highest value of 99.1%. Meanwhile, training and validation loss gradually decline and are close to zero at the end.

### 5.4. Qualitative Evaluations

**Figure 10:**
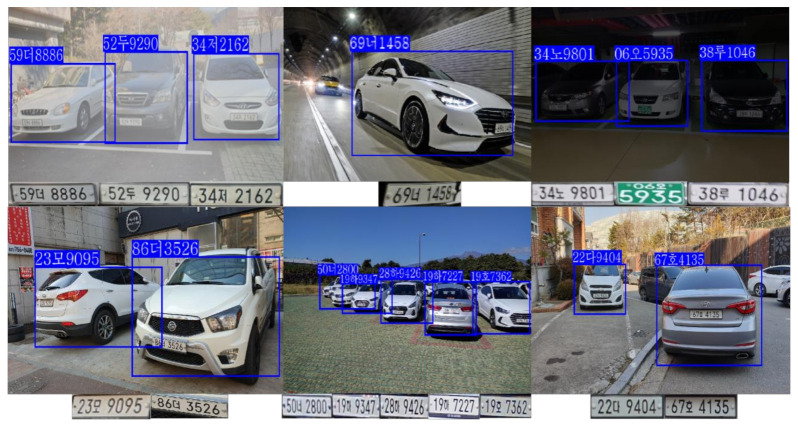
Different LP templates were detected and recognized correctly under foggy and dark environments and at different angles, such as the back side, front side, left side, and right side.

**Figure 11:**
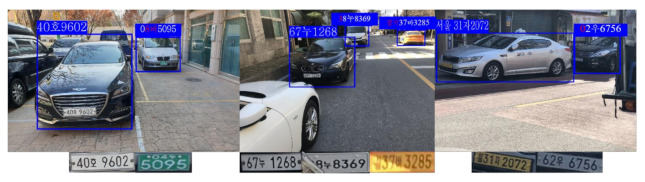
Some wrong recognition cases (the red characters: wrong recognition).

In this system, some images that consist of various LPs are presented to prove the robustness of the LPR system by changing different variations (e.g., weather, blurriness, illumination, and angle). Figure 10 and Figure 11 show qualitative evaluations of the proposed system on the testing set. Lots of LPs can be recognized well in different scenes, in particular when the obtained image is extremely dim or under bad weather. However, there also exist some bad examples where the LP that is very far from the camera is recognized mistakenly or fails to recognize the LP that is blocked by an adjacent car.

In this study, the recognition accuracy and the processing time are regarded as vital factors to evaluate the proposed methods. 200 images collected from the parking lot are used to test the performances of the two approaches. The overall performance of vehicle detection, license plate detection, and the two LPR approaches are shown in Table 4. It can be seen that the LPR system can detect vehicles and LPs in a short time. The processing speed of the W-LPR method can reach 0.062 s per image, which is 0.064 s faster than the SC-LPR method for the exact same image. In terms of recognition accuracy, the W-LPR method can achieve a little higher recognition accuracy than the SC-LPR method.

In addition, it is challenging to conduct a fair comparison with many methods due to the unavailability of Korean public LP data and the commercial nature of most LPR systems. In this section, the performance of the proposed LPR system is evaluated and compared with other state-of-the-art LPR systems proposed in [13,32] by using the same dataset (KarPlate) [32]. The detected LP is regarded as a corrected prediction when the Intersection over Union (IOU) is equal to 0.5. The accuracy of LPR indicates the percentage of correctly predicted LPs in the whole test set. If both sequences and characters in the LP are recognized correctly, then the recognition result of the LP is correct. The accuracy of EER represented the overall accuracy of the LPR system. When the system can detect the vehicle and LP correctly and recognize the LP correctly, the EER result of a single image is correct. 

As shown in Table 5, the proposed methods achieve better recognition results than their result on both the LPR subset and EER subset. However, our LPD precision is lower than the result of the system presented in [32]. That is because the detection of the LP in our LPR system includes two parts: vehicle detection and license plate detection. In the process of vehicle detection, the LP area of several images in the dataset is not completely acquired, which affects the detection of the license plate. In terms of the processing speed, the proposed W-LPR method in this study takes 0.069 s per image based on the same dataset, which is 0.003 s slower than ALPR. The reason is that the effective speed of the graphics card (Tian X Pascal) used in their experiment is 68% faster than ours (GTX Titan X). 

## 6. Conclusions

In this study, based on deep learning, an LPR system was proposed to identify various types of Korean license plates. There are two sets of data for different LPR methods. A total of 510,500 LP images were obtained for the W-LPR method, of which 500,000 synthetic images were generated by a python script, and 10,500 real images were taken in different scenes. 216,000 images consisting of various characters were generated to feed the model of the SC-LPR method by using the proposed character segmentation method. In the phase of image preprocessing, three technologies were applied to improve the quality of the input image. In addition, two customized methods were proposed to recognize Korean license plates. A model combining CNN and RNN was used to train the images of whole LPs in the W-LPR method. The SC-LPR method was divided into two steps, the characters on the LP were segmented, and then the character was identified by a proposed CNN model. Experiment results show both methods achieved similar good recognition accuracy, while the processing speed of the W-LPR method for each image is faster than the SC-LPR method. In addition, the current LPR system is based on multiple deep learning models, which takes a lot of storage memory.

In the future, more attention will be put on reducing the size of the LPR system, because the size of the current LPR system is large, which is not suitable for installation in mobile applications. Moreover, more images collected from various countries need to be added to the research and improve the overall recognition accuracy such that the performance of the LPR system can be widely used in different countries.

## Figures and Tables

**Table 1:** Detailed information of license plate format in South Korea (# stands for a digit).

Private Vehicles			Commercial Vehicles		
Format	## Korean Character ####		Format	Province/City ## Korean Character ####	
	### Korean Character ####				
Element	Symbol	Meaning	Element	Symbol	Meaning
**##**	01–69	Typical passenger vehicles	**##**	11–69	Taxis
	70–79	Vans, coaches, recreational vehicles		70–79	Vans and buses
	80–97	Trucks		80–97	Trucks
	98–99	Specialized vehicles		98–99	Specialized vehicles
**Korean character**	가, 나, 다, 라, 마, 거, 너, 더, 러, 머, 버, 서, 어, 저, 고, 노, 도, 로, 모, 보, 소, 오, 조, 구, 누, 두, 루, 무, 부, 수, 우, 주	Private vehicles	**Province/City**	강원, 경기, 경남, 경북, 광주, 대구, 대전, 부산, 서울, 세종, 울산, 인천,전남, 전북, 제주, 충남, 충북	Province or city
	허,하,호	Rental cars			
**###**	100–699	Typical passenger vehicles	**Korean character**	바, 사, 아, 자	Taxi/bus
				배	Delivery van/truck
**####**	1000–9999	Regardless of the car type	**####**	1000–9999	Regardless of the car type

**Table 2:** The structure of the character recognition model.

Layer	Type	Number of Feature Maps & Size	Kernel
1	Input	1 map with 32 × 32 neurons	-
2	Convolution	16 maps with 30 × 30 neurons	3 × 3
3	Max pooling	16 maps with 15 × 15 neurons	2 × 2
4	Convolution	32 maps with 13 × 13 neurons	3 × 3
5	Max pooling	32 maps with 6 × 6 neurons	2 × 2
6	Convolution	32 maps with 4 × 4 neurons	3 × 3
7	Fully-connected	512 neurons	-
8	Fully-connected	128 neurons	-
9	Softmax	70 neurons	-

**Table 3:** The performances of the three image preprocessing technologies, which include defogging, low-light enhancement, and super-resolution. The wrong characters are marked in red.

		Process Time (s)	Image	Recognition Results (W-LPR)	Recognition Results (SC-LPR)	Ground Truth
Defogging	Before	0.014	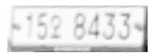	15모8433	16모8438	15오8433
	After		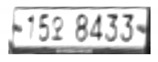	15오8433	15오8433	
Low-light enhancement	Before	0.002	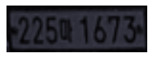	225아7673	226아1613	225마1673
	After		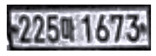	225아1673	225마1673	
Super-resolution	Before	0.049	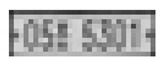	05로5601	05고6801	05조5301
	After		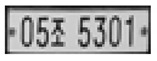	05조5301	05조5301	

**Table 4:** The processing time and the recognition accuracy of the two approaches.

	Time (s)				Recognition Accuracy (%)
	VD	LPD	LPR	Total	
W-LPR	0.015	0.02	0.027	0.062	99.5
SC-LPR	0.015	0.02	0.091	0.126	98.9

**Table 5:** The performance comparison of different methods that were implemented in this study, which include LPD, LPR, and EER.

Approach	The Precision of LPD	The Accuracy of LPR	The Accuracy of EER
OpenALPR [13]	-	-	97.95
ALPR [32]	100.0	98.59	98.17
W-LPR	99.18	99.18	98.94
SC-LPR	99.18	98.80	98.25
